# BMSCs and Osteoblast-Engineered ECM Synergetically Promotes Osteogenesis and Angiogenesis in an Ectopic Bone Formation Model

**DOI:** 10.3389/fbioe.2022.818191

**Published:** 2022-01-21

**Authors:** Chi Zhang, Dongdong Xia, Jiajing Li, Yanan Zheng, Bowen Weng, Haijiao Mao, Jing Mei, Tao Wu, Mei Li, Jiyuan Zhao

**Affiliations:** ^1^ Zhejiang Key Laboratory of Pathophysiology, School of Medicine, Ningbo University, Ningbo, China; ^2^ Medical Research Center, Ningbo City First Hospital, Ningbo, China; ^3^ Orthopedic Department, Ningbo City First Hospital, Ningbo, China; ^4^ Department of Orthopaedic Surgery, the Affiliated Hospital of Medical School, Ningbo University, Ningbo, China; ^5^ Cardiovascular Center, the Affiliated Hospital of Medical School, Ningbo University, Ningbo, China; ^6^ Ningbo Institute of Medical Sciences, Ningbo, China

**Keywords:** bone mesenchymal stem cells, extracellular matrix modification, ectopic bone formation model, osteogenesis, angiogenesis

## Abstract

Bone mesenchymal stem cells (BMSCs) have been extensively used in bone tissue engineering because of their potential to differentiate into multiple cells, secrete paracrine factors, and attenuate immune responses. Biomaterials are essential for the residence and activities of BMSCs after implantation *in vivo*. Recently, extracellular matrix (ECM) modification with a favorable regenerative microenvironment has been demonstrated to be a promising approach for cellular activities and bone regeneration. The aim of the present study was to evaluate the effects of BMSCs combined with cell-engineered ECM scaffolds on osteogenesis and angiogenesis *in vivo*. The ECM scaffolds were generated by osteoblasts on the small intestinal submucosa (SIS) under treatment with calcium (Ca)-enriched medium and icariin (Ic) after decellularization. In a mouse ectopic bone formation model, the SIS scaffolds were demonstrated to reduce the immune response, and lower the levels of immune cells compared with those in the sham group. Ca/Ic-ECM modification inhibited the degradation of the SIS scaffolds *in vivo*. The generated Ca/Ic-SIS scaffolds ectopically promoted osteogenesis according to the results of micro-CT and histological staining. Moreover, BMSCs on Ca/Ic-SIS further increased the bone volume percentage (BV/TV) and bone density. Moreover, angiogenesis was also enhanced by the Ca/Ic-SIS scaffolds, resulting in the highest levels of neovascularization according to the data ofCD31 staining. In conclusion, osteoblast-engineered ECM under directional induction is a promising strategy to modify biomaterials for osteogenesis and angiogenesis. BMSCs synergetically improve the properties of ECM constructs, which may contribute to the repair of large bone defects.

**GRAPHICAL ABSTRACT:**
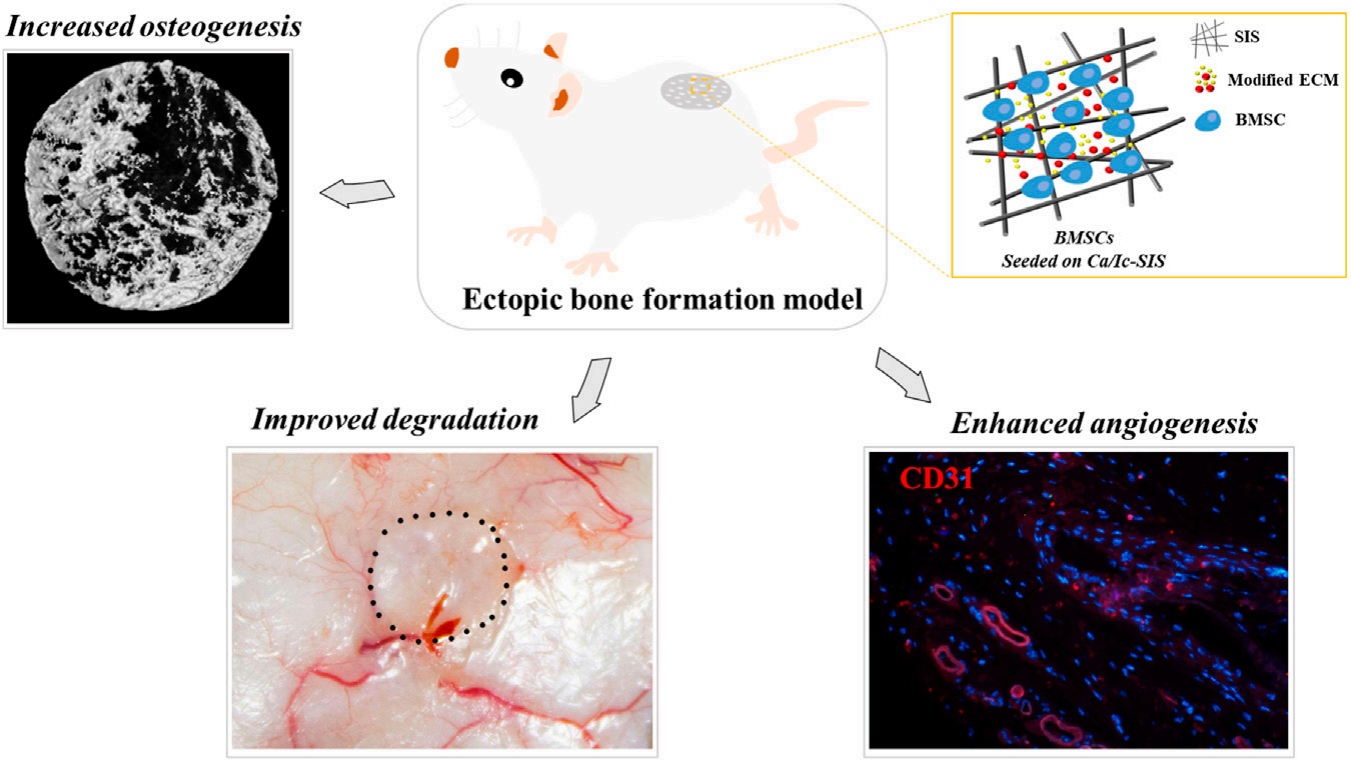


## Introduction

Reconstruction of large bone defects caused by trauma or disease has always been a major challenge in the clinic (Chen et al., 2021). Autologous bone grafts are considered the “gold standard”; however, limited availability and donor site morbidity associated with auto grafts restricts their clinical applications (Ghate and Cui, 2017; Yan et al., 2020). Tissue engineering was developed as a promising method for graft-based bone repair and regeneration to solve this problem. Traditional tissue engineering involves scaffolds, seeding cells, and growth factors; scaffolds are the key components that provide structural and mechanical support for the cells (Roseti et al., 2017). Moreover, scaffolds with a proper microenvironment can determine cell fate during bone regeneration. Unlike metal, ceramic, and polymer scaffolds, extracellular matrix (ECM) is more beneficial for cellular activities directed toward tissue reconstruction because of mimetic tissue niches with multiple chemical and physical cues (Sun et al., 2021). Therefore, in recent decades, ECM-based scaffolds have attracted the attention of investigators as a new generation of biomaterials for tissue engineering (Mansour et al., 2017; Shang et al., 2021).

The small intestinal submucosa (SIS) is a native ECM scaffold from the submucosal layer of the porcine jejunum obtained after decellularization that has been approved by the FDA in the clinic (Kropp et al., 1995). Acellular SIS preserves three-dimensional (3D) architecture and ECM components, including collagen (more than 90%), glycosaminoglycans, and fibronectin. Additionally, a wide variety of cytokines have been detected in the ECM, such as basic fibroblast growth factor (bFGF), transforming growth factor-β (TGF-β), and vascular endothelial growth factor (VEGF) (Hodde et al., 1996; McPherson and Badylak, 1998). Moreover, xenogeneic SIS-based biomaterials for transplantation are known as absorbable materials and elicit reduced inflammatory immune responses (Andrée et al., 2013). Increasing evidences have demonstrated the advantages of SIS in tissue engineering for reconstruction of various tissues, such as blood vessels (Kakisis et al., 2005), bone (Kim et al., 2010), cartilage (Le Visage et al., 2006), bladder, and ureter (Kropp et al., 1995; Desgrandchamps, 2000). Our previous studies mainly focused on the application of the SIS scaffolds in bone tissue engineering and regeneration. SIS was demonstrated to be a potential osteoconductive and osteoinductive biomaterial with excellent biocompatibility that recruited various key cells (BMSCs, osteoblasts, and fibroblasts) and promoted their proliferation, osteogenesis, vascularization, and bone regeneration (Li et al., 2017b). However, limited new bone (∼14%) was formed until 8 weeks after transplantation in a critical-size mouse calvarial defect model. The results may be explained by soft tissue origin of SIS with different tissue microenvironments, which limited the application of SIS for bone regeneration. Thus, additional osteoinductive biomolecules (e.g., bone tissue components) should be introduced to modify SIS to improve its osteoinductivity (Zhao et al., 2020).

Along with the development of tissue engineering, numerous methods have been created to modify the scaffolds and improve the functions of the implants. These methods include the coating of the scaffold surfaces that has been demonstrated to greatly influence the biocompatibility and biological activities of underlying biomaterials (Zhao et al., 2020). Recently, cell-derived ECM (CDM) coatings have attracted attentions of the researchers. Similar to decellularized tissues, CDM provides complex components of natural ECM with a 3D structure and represents unique compositions and niches of variable cell origin (Cheng et al., 2014). Fu et al. (2018) demonstrated that coating of the PLLA NF surface with MC3T3-E1 CDM enhanced the adhesion of mouse bone marrow stromal cells (mBMSCs), supported cell proliferation, and promoted osteogenic differentiation of mBMSCs. Feng et al. (2020) fabricated the CDM sheet-implant complexes using a combination of rat BMSCs (rBMSCs) CDM sheets with the sandblasted, large-grit, and acid-etched (SLA) implants. The generated CDM sheet-implant complexes were demonstrated to have low immunogenicity and resulted in superior new bone formation *in vivo*. Our previous studies also used MC3T3-E1 CDM to modify the SIS scaffolds, and the generated ECM-SIS scaffolds were proven to enhance bone repair *in situ* in a mouse calvarial defect model (Zhang et al., 2017; Li et al., 2018). Interestingly, we also demonstrated that drug-derived CDM is a promising novel method to deliver drugs to improve mineral deposition and bone formation induced by the SIS scaffolds (Li et al., 2017a; Li et al., 2017c).

Besides the scaffolds, seeding cells are also essential to promote the reconstruction, and regeneration of tissue defects. BMSCs are a well-characterized population of adult stem cells with capabilities for self-renewal and multipotent differentiation (Shakouri-Motlagh et al., 2017). Preosteogenic chondroblasts and osteoblasts are two major pathways of BMSCs differentiation that contribute to bone regeneration. Along with the development of tissue engineering techniques, BMSCs transplantation has been used in the clinic and has been demonstrated to enhance tissue regeneration, especially in the vasculature (Gong and Niklason, 2008), cartilage reconstruction (Bernardo et al., 2007), and new bone formation (Yamada et al., 2004). We have previously reported that decellularized osteogenic ECM is beneficial for BMSCs expansion and mineralization *in vitro* (Li et al., 2019). However, whether SIS scaffolds coated with osteogenic ECM with BMSCs facilitate bone regeneration *in vivo* was not investigated. Animal models of ectopic bone formation have unique advantages over *in situ* bone environments, including a relative lack of bone cytokine stimulation, and cell-to-cell interaction with host bone-forming cells. A wide variety of ectopic models have been used for experimentation, including subcutaneous, intramuscular, and kidney capsule transplantation. Subcutaneous implantation is the simplest method and is extremely useful for evaluation of growth factors, novel osteoinductive biomaterials, and engrafted stem cells (Scott et al., 2012).

In summary, our previous study developed a bone mimetic ECM construct guided by osteoblasts under icariin treatment in calcium-enriched medium, which was proven to facilitate bone regeneration in a mouse calvarial defect model (Li et al., 2018). Thus, the present study aimed to expand the evaluation and application of the bone mimetic ECM construct for bone regeneration and angiogenesis in combination with BMSCs. A mouse ectopic osteogenesis model was introduced to assess the effects *in vivo*. The raw SIS, regular ECM-modified SIS, and Ca/Ic ECM-modified SIS scaffolds were subcutaneously implanted into ICR mice with or without BMSCs. The immune response, scaffold biodegradability, osteogenic properties, and blood vessel formation were studied.

## Materials and Methods

### Ethics Statement

All mice used in the present study were purchased from Charles River (Zhejiang, China) and housed in the Animal Center of Ningbo University. All experimental procedures involving animals were conducted in compliance with Chinese legislation regarding the use and care of laboratory animals and were approved by the Animal Care and Use Committee of Ningbo University.

### Isolation and Culture of the Cells

MC3T3-E1 cells were purchased from the Type Culture Collection of the Chinese Academy of Sciences (Shanghai, China) and cultured in αMEM medium (Gibco, Carlsbad, CA, United States) supplemented with 10% fetal bovine serum (FBS) (Gibco). BMSCs were isolated from ICR mice at the age of 8 weeks according to our previous reports (Zhang et al., 2018). Briefly, the mice were sacrificed by cervical dislocation and soaked in 70% (v/v) ethanol for 3 min, and then were transferred to new 100-mm cell culture dishes. The skin, muscles, ligaments and tendons were carefully disassociated from tibias, and femurs of hind limbs. Tibias and femurs were dissected and transferred to a new dish and washed twice with PBS. After dissection of the two ends of the bones, the marrow was slowly flushed out via a needle until the bones became pale. The BMSCs pool were filtered through a 70 μm strainer and cultured in DMEM (Gibco) supplemented with 10% FBS (Si Jiqing, China). Passage 3 BMSCs were collected for the experiments.

### Preparation of Biomimetic ECM-Modified SIS Scaffold

Lyophilized SIS scaffolds were kindly supplied by Cook Biotech Inc. (West Lafayette, Indiana). The SIS scaffolds were cut into the round constructs 5 mm in diameter for *in vivo* experiments by a biopsy punch (Miltex, Loznica, and Serbia) as described previously (Li et al., 2017a; Li et al., 2017c). MC3T3-E1 cells were seeded on the SIS scaffolds for 4 weeks to obtain abundant ECM. For Ca/Ic-SIS preparation, the concentrations of CaCl_2_ and icariin were selected as described in previous studies (Takagishi et al., 2006; Zhao et al., 2009). Briefly, the scaffolds were rehydrated in complete culture medium at 37°C for at least 24 h before cell culture. Approximately 5 × 10^4^ MC3T3-E1 cells were dropped on the SIS scaffolds. On the next day, the SIS scaffolds with the cells were transferred to new 96-well plates and cultured in regular medium or induction medium (containing 10 mM CaCl_2_ and 10 μM icariin) for 4 weeks. The medium was changed every other day. Decellularization was performed as reported previously (Pati et al., 2015; Li et al., 2018). Briefly, the samples were subjected to three freeze-thaw cycles in a −80°C freezer and 37°C water bath (30 min for each step). After each thawing step, the scaffolds were rinsed in sterile phosphate-buffered saline (PBS) to remove cellular components. After decellularization, two kinds of biomimetic ECM-modified SIS were generated and stored at −80°C before use.

### Immune Response of the SIS Scaffolds After Subcutaneous Transplantation in Mice

Eight-week-old male ICR mice weighing 20–25 g were used to assess the immune response of the scaffolds after implantation *in vivo*. The mice were randomly divided into four groups (Ca/Ic-SIS group, ECM-SIS group, SIS group, and sham group). The scaffolds (Φ5 mm) were surgically implanted into subcutaneous sites of ICR mice. The sham group undergoing surgical operation without scaffold implantation was considered a control. Operations were performed under general anesthesia by intraperitoneal injection of 1.2% avertin–PBS solution (20–25 μl/g) on a super clean bench. The blood from the tail vein (20 μl) was collected and mixed with 1.2 ml of a diluent (Bolai Biotech Inc., Jinan, China). The levels of white blood cells (WBCs), monocytes (MONs), and lymphocytes (LYMs) were detected by a hemocyte analyzer (Boehringer MannheimBM830, China) at various time points (1, 2, 3, 5, 7, 9, 11, 13, 15, 18, 21, and 24 days). Four replicates from each group at each time point were assayed, and the results were averaged.

### *In vivo* Degradation Assay of the SIS Scaffolds

The scaffolds (Φ5 mm) were split into the following six groups: SIS, ECM-SIS, Ca/Ic-SIS, SIS + BMSCs, ECM-SIS + BMSCs, and Ca/Ic-SIS + BMSCs, with four animals per group. All groups were individually surgically implanted into subcutaneous sites of ICR mice. Degradation of the scaffolds *in vivo* was detected after 8 weeks by gross view. The implant areas were measured by ImageJ.

### Effect of the Ca/Ic-SIS Scaffolds With BMSCs on Ectopic Osteogenesis

A mouse subcutaneous transplantation model was used to assess ectopic osteogenesis and vessel formation induced by the Ca/Ic ECM-modified SIS scaffolds. The animals were randomly divided into the following six treatment groups (Φ5 mm, *n* = 4): SIS, ECM-SIS, Ca/Ic-SIS, SIS + BMSCs, ECM-SIS + BMSCs, and Ca/Ic-SIS + BMSCs. In the groups treated with BMSCs, 1 × 10^5^ cells were seeded on the raw SIS, ECM-SIS, or Ca/Ic-SIS scaffolds in basal medium without osteogenic inductive factors. After incubation for 12 h at 37°C to allow cell attachment, the cell/scaffold composites were surgically implanted into subcutaneous sites of ICR mice. Operations were performed under general anesthesia by intraperitoneal injection of 1.2% avertin–PBS solution (20–25 μl/g) on a super clean bench. All animals were sacrificed at 8 weeks after the surgery.

### Micro-CT Analysis

After 8 weeks, the implants were harvested and assessed via radiographic analysis using a micro-CT scanner (NEMO Micro-CT; PINGSENG Healthcare, China). The scanned CT images of the specimens were acquired at a resolution of 50 mm (achieved using 90 kV and 60 μA) and three-dimensionally (3D) reconstructed using computer software (Recon; PINGSENG, Shanghai, and China). The regenerated bone volume and bone density in the defect site, and tissue density in the region of interest (ROI) of all groups were calculated based on 3D reconstructed images using Mimics (Materialise).

### Sample Harvesting and Histological Staining

After micro-CT scanning, the samples were fixed in 10% formalin for 24 h. The samples were then rinsed with PBS, decalcified in 10% (w/v) sodium citrate/22.5% (v/v) formic acid (Morse’s solution) for 2 days, neutralized with 5% sodium sulfate for 6 h, and washed with water for 6 h. The samples were then dehydrated, embedded in paraffin, and sectioned (5 μm). Hematoxylin and eosin (H&E) and Masson’s trichrome staining (MTS) were performed according to the standard procedures for histological analysis as reported previously (Li et al., 2017b; Zhang et al., 2017).

### Effect of the Ca/Ic-SIS Scaffolds With BMSCs on Angiogenesis *in vivo*

Evaluation of the effects of the implants with BMSCs on angiogenesis was performed using H&E and immunofluorescence (IF) staining. IF staining was performed following the protocol of Cell Signaling Technology (CST). Briefly, the samples were fixed in 4% formaldehyde for 15 min at room temperature and rinsed with PBS. Before blocking with normal goat serum, the sections were incubated in methanol for 10 min at −20°C. The samples were incubated with a CD31 antibody (1:200, CST) in 1% BSA/0.3% Triton X-100/PBS at 4°C overnight. A secondary antibody was conjugated with Alexa Fluor® 555 (CST, Shanghai, China). The nuclei were stained with DAPI, and the samples were mounted in anti-fade solution. The samples were imaged under a fluorescence microscope (OlympusIX71/IX51, Japan).

The effect of angiogenesis induced by the scaffolds and/or BMSCs was indicated by the area percentage of neovascularization divided by the area of tissue sections in the same image at same magnification. The areas of the neovascularization and tissue sections were measured by ImageJ based on IF staining of CD31. Four mice were assayed for each group, and at least 5 sections at various sites were measured for each mouse.

### Statistical Analysis

Statistical analysis was performed using SPSS Statistics 23.0 (IBM Corporation, Armonk, NY, United States). The ratio of the new vessels per implant area was quantified based on IF staining for CD31. The results are presented as the median (with range), and statistical analysis of the results was performed by the Mann-Whitney U test. Other quantitative data are expressed as the mean ± standard deviation. Statistical significance was determined using a two-tailed unpaired t-test (two-group comparison) or one-way analysis of variance (ANOVA) followed by a post hoc test (multigroup comparison). A *p* value of <0.05 was considered statistically significant.

## Results

### Immune Response of the Scaffolds After Transplantation

**FIGURE 1:**
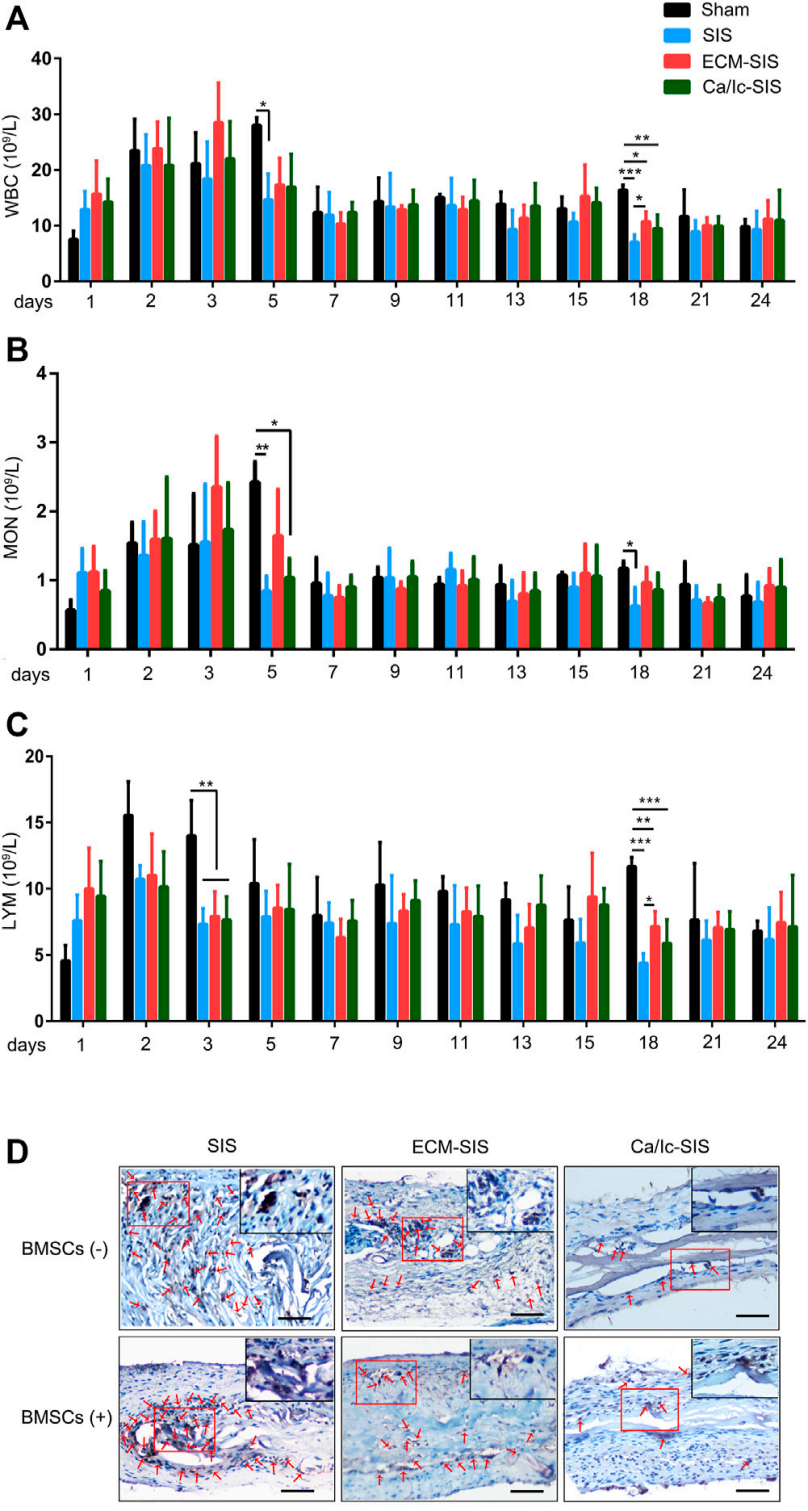
Histogram of **(A)** white blood cells (WBCs), **(B)** monocytes (MONs), and **(C)** lymphocytes (LYMs) counts as a function of time (days) after the operation. Mean +S.D. (*n* = 4). *, *p* < 0.05; **, *p* < 0.01, and ***, *p* < 0.001. **(D)** IHC staining of CD68 in the six scaffolds (SIS, ECM-SIS, Ca/Ic-SIS, SIS + BMSCs, ECM-SIS + BMSCs, and Ca/Ic-SIS + BMSCs) at 8 weeks after implantation.

A reduction in the immune response is vital for biomaterial transplantation in the clinic. In the present study, the levels of white blood cells (WBCs), monocytes (MONs), and lymphocytes (LYMs) in the blood were used to assess inflammatory reactions after implantation of the SIS-based scaffolds (Figure 1). During the first 3 days, the numbers of WBCs and MONs were increased in all groups because of surgical operation (Figure 1A,B). However, significant differences in these two indexes between the sham and SIS implantation groups were detected on day 5. The numbers of inflammatory cells were continuously increased in the sham group, but decreased in the SIS-implanted groups. Significant differences in the number of LYMs were manifested as lower values in the SIS-implanted groups up to day 3 compared with those in the sham group (Figure 1C). The results indicated that the SIS scaffolds not only had low immunogenicity but also reduced the immune response caused by surgical operation. Moreover, expression of CD68 (an macrophage marker) in the tissues at 8 weeks after implantation was detected by IHC staining. As shown in Figure 1D, CD68 expression was down-regulated in ECM-modified SIS (especially Ca/Ic-SIS) implants, compared with the raw SIS implants.

### Ca/Ic ECM Modification Inhibited the Degradation of the SIS Scaffolds *in vivo*

**FIGURE 2:**
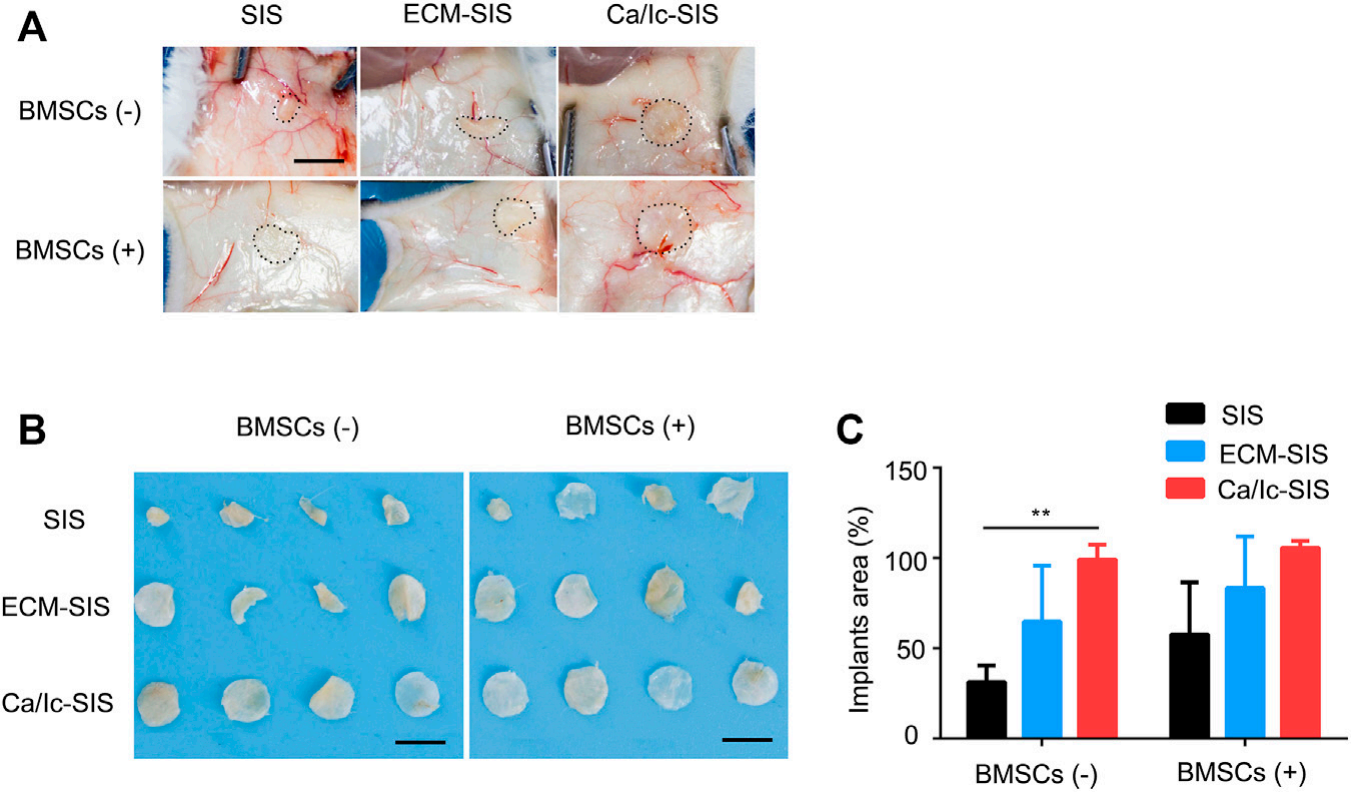
Degradation profiles of the SIS, ECM-SIS, and Ca/Ic-SIS scaffolds (seeded with or without BMSCs) after 8 weeks. Gross views *in vivo* **(A)** and *in vitro* **(B)**. **(C)** Quantitative analysis of the degradation of the six scaffolds (SIS, ECM-SIS, Ca/Ic-SIS, SIS + BMSCs, ECM-SIS + BMSCs, and Ca/Ic-SIS + BMSCs). The raw SIS scaffolds were significantly degraded into a small size, and no detectable degradation was observed in the case of the Ca/Ic-SIS + BMSCs scaffolds. Mean +S.D. (*n* = 4). **, *p* < 0.01. Scale bar: 5 mm.

The SIS and ECM-modified SIS scaffolds with or without BMSCs were prepared as described in the section of Materials and methods and surgically implanted into subcutaneous sites of ICR mice. SIS is a natural ECM biomaterial that can be degraded *in vivo*. However, rapid degradation within a short time had an actual adverse effect on tissue reconstruction due to insufficient support. As shown in Figure 2, the raw SIS scaffolds were clearly degraded into a smaller area, compared with the scaffolds in other groups at 8 weeks after transplantation. The shape of the Ca/Ic ECM-modified SIS scaffolds was maintained regardless of the presence of BMSCs (Figure 2A,B). The area of the residual scaffolds was measured, and a significant difference between SIS and Ca/Ic SIS was detected in the absence of BMSCs (Figure 2C). After BMSCs seeding, the average area of the SIS and ECM-SIS scaffolds was smaller than that of the Ca/Ic SIS scaffolds. However, no significant difference was observed among the groups. No statistical difference may be caused by the high deviation in the SIS and ECM-SIS scaffolds, which may be attributed by BMSCs activities. Therefore, the results demonstrated that Ca/Ic modification was able to slow down the degradation of the SIS scaffolds *in vivo*.

### Ca/Ic ECM Modification Promoted Ectopic Bone Formation

**FIGURE 3:**
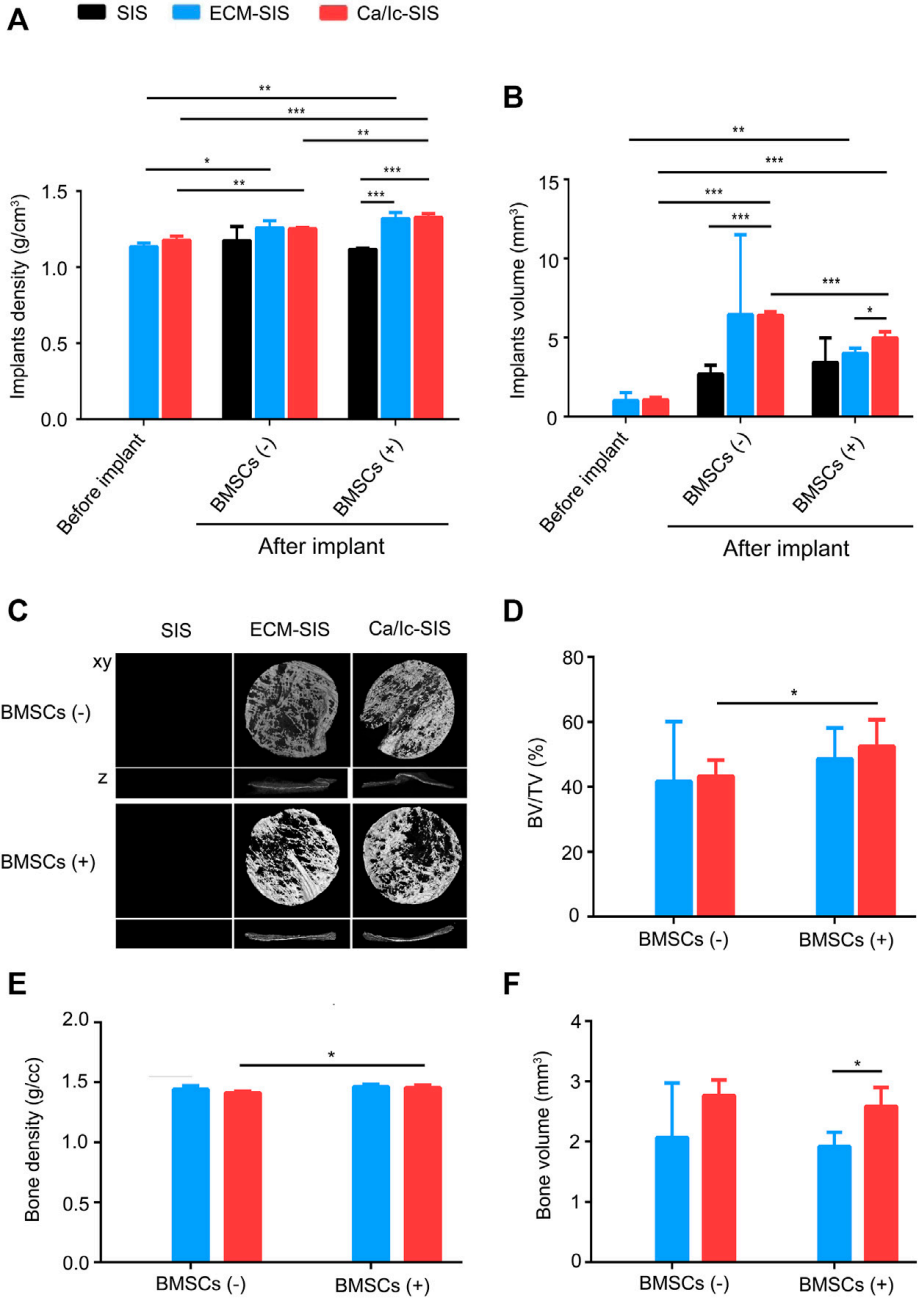
Micro-CT evaluation and ectopic bone formation induced by ECM-SIS and Ca/Ic-SIS (with or without BMSCs) 8 weeks after subcutaneous implantation in mice. The density **(A)** and volume **(B)** of the scaffolds before or after the implantation were analyzed by micro-CT. **(C)** Representative 3D reconstructed micro-CT images 8 weeks after the implantation. The new bone formation ratio **(D)**, regenerated bone density **(E)**, and bone volume in the region of interest (ROI) **(F)** of each group were calculated based on 3D reconstructed images using Mimics (Materialise). Mean +S.D. (*n* = 3).*, *p* < 0.05, **, *p* < 0.01, and ***, *p* < 0.001.

Micro-CT was used to analyze ectopic bone formation at 8 weeks after implantation of the SIS-based scaffolds. Initially, the whole tissues with the scaffolds were assessed before and after the transplantation. As shown in Figure 3A,B, the density (A) and volume (B) of the ECM-SIS and Ca/Ic-SIS scaffolds were significantly higher than those of the raw SIS scaffolds before the transplantation. After 8 weeks *in vivo*, the volume of the Ca/Ic-SIS scaffolds was the highest among the three kinds of the implants with or without BMSCs, which was consistent with the gross view shown in Figure 2. The results further confirmed that Ca/Ic modification was able to slow down the degradation of the SIS scaffolds *in vivo*. BMSCs further increased the density of the Ca/Ic-SIS implants after the transplantation (Figure 3A). Then, the bone volume and density were evaluated (Figures 3C–F). In the raw SIS groups, no new bone was detected after transplantation no matter with or without BMSCs in the ectopic bone model (Figure 3C), while newly formed bone was apparent in the ECM-modified SIS groups (ECM-SIS and Ca/Ic-SIS) regardless of the absence or presence of BMSCs (Figure 3C). Moreover, the groups with the Ca/Ic-SIS implants had a higher level of newly formed bone than other groups, as assessed by BV/TV (Figure 3D) and bone volume measurements (Figure 3F). BMSCs further increased BV/TV (Figure 3D) and bone density (Figure 3E) of the Ca/Ic-SIS implants. BV/TV and bone volume were used to assess the quantity of regenerated bone, while bone density reflected the quality of regenerated bone.

**FIGURE 4:**
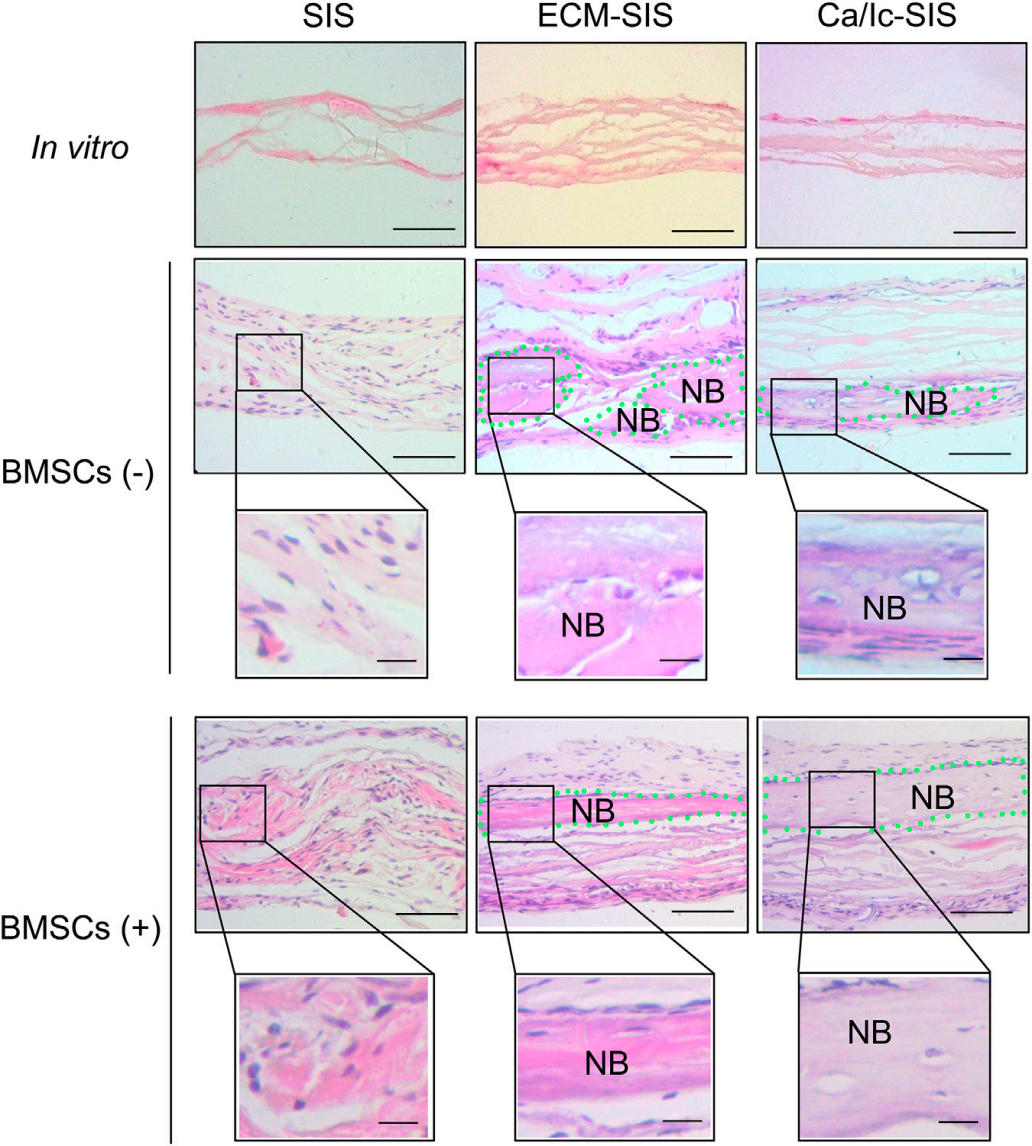
Representative histological images (hematoxylin-eosin staining) showed various bone regenerative effects of the six scaffolds (SIS, ECM-SIS, Ca/Ic-SIS, SIS + BMSCs, ECM-SIS + BMSCs, and Ca/Ic-SIS + BMSCs) after 8 weeks. Considerable formation of the new bone was detected in the ECM-SIS and Ca/Ic-SIS groups (seeded with or without BMSCs). Green dotted region represented newly formed bone (NB). Scale bar: 100 μm.

**FIGURE 5:**
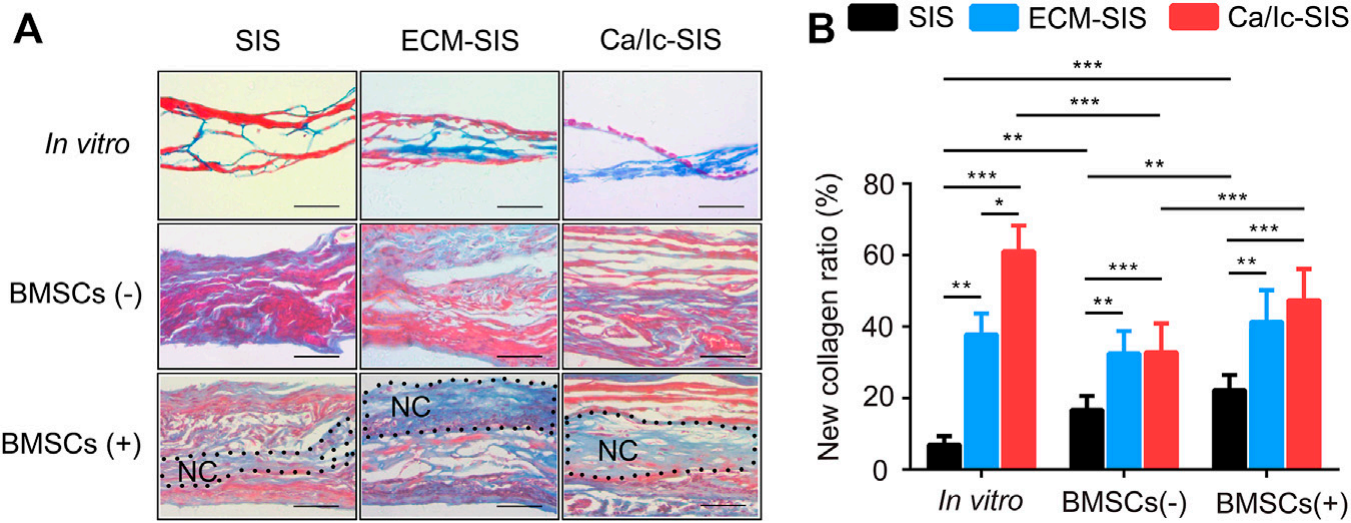
MTS staining of the cross-sections of the scaffold implants. **(A)** Representative images of all groups are shown after 8 weeks. Collagen of the new bone matrix was stained as blue. **(B)** Collagen area with blue color and the whole tissue area were measured by ImageJ software, and the ratio was calculated. Five sections were measured for each mouse and four mice were analyzed for each group. *, *p* < 0.05, **, *p* < 0.01 and ***, and *p* < 0.001. Black dotted region represented newly formed collagen (NC). Scale bar: 100 μm.

Histological analyses of tissue samples at 8 weeks after transplantation were carried out to characterize new bone formed in the ECM-modified SIS scaffolds (Figures 4, 5). No significant inflammatory or immune response was detected by H&E staining in the groups subcutaneously implanted with the raw SIS and ECM-modified SIS scaffolds (Figure 4). Cell recruitment to the scaffolds in all implants confirmed biocompatibility of the SIS scaffolds. All scaffolds became thicker in the post-implantation groups compared with those in the pre-implantation groups, indicating that tissue cells grew into the scaffolds. Moreover, bone-like tissues were detected in both ECM-SIS and Ca/Ic-SIS implants; however, these tissues were rarely detected in the raw SIS implants. BMSCs accelerated new bone formation in both ECM-modified SIS implants, particularly in the Ca/Ic-SIS implants. Thus, the histological results were consistent with the data of micro-CT scanning. In addition to H&E staining, Masson’s trichrome staining (MTS) was used to analyze newly ectopically formed bone (Figure 5A). Statistical analysis showed that the ratio of new regenerative collagen were higher in the ECM-modified SIS implants with BMSCs than those in the groups with raw SIS implants and no BMSCs seeding (Figure 5B).

### Evaluation of Angiogenesis

**FIGURE 6:**
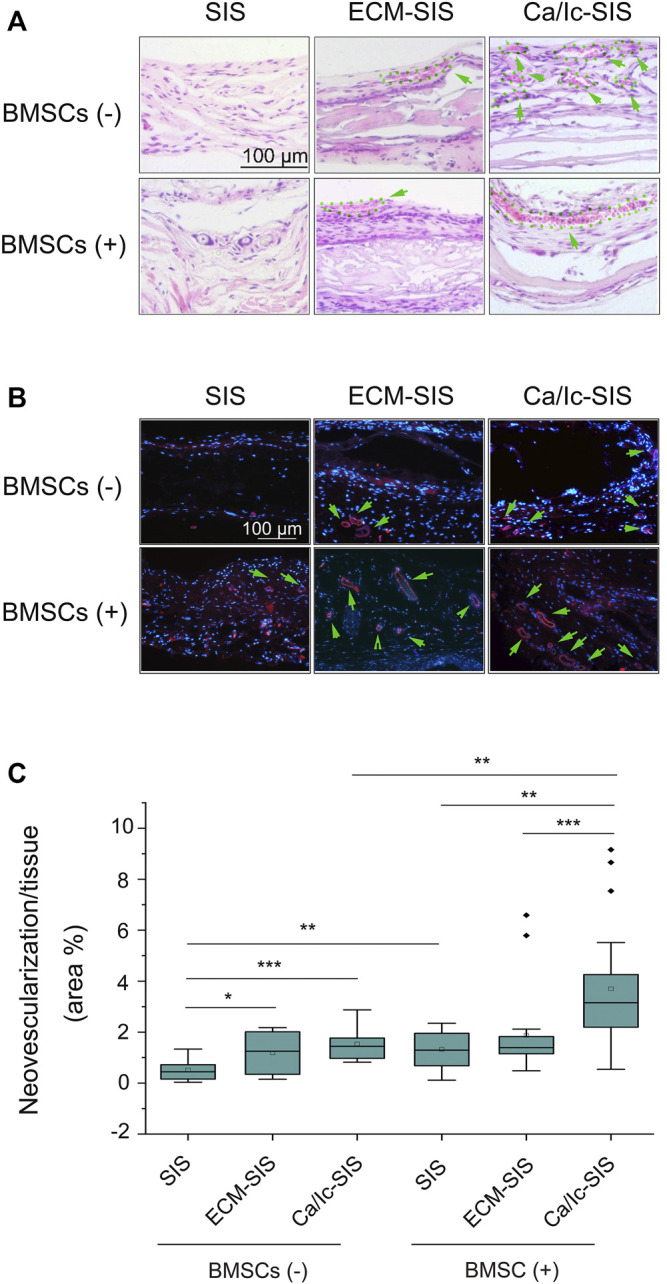
**(A)** Histological images (H&E staining) of various vascular-like structures in the groups. **(B)** Immunofluorescence staining for CD31 in the scaffolds 8 weeks after the implantation *in vivo*. New vessels were identified by positive CD31 staining (red), and the nuclei were stained with DAPI (blue). **(C)** Box plots showing the area percentage of new vessel formation in the SIS, ECM-SIS, and Ca/Ic-SIS (with and without seeding BMSCs) scaffolds. The area of neovascularization and implanted tissue was quantified by ImageJ software. Five sections were measured for each mouse and four mice were analyzed for each group. *, *p* < 0.05, **, *p* < 0.01, and ***, *p* < 0.001. Scale bar: 100 μm. Green arrows directed blood vessels and the corresponding regions were circled by green dotted lines.

Angiogenesis is essential for large bone regeneration, and numerous efforts have been made to improve angiogenic properties of biomaterials. In the present study, neovascularization with red blood cells were detected in two kinds of ECM-modified SIS scaffolds (ECM-SIS and Ca/Ic-SIS) with or without BMSCs according to the data of H&E staining (Figure 6A). IF staining for CD31was used for specific detection of the vessels (Figure 6B), and angiogenesis induced by the scaffolds and/or BMSCs was quantified (Figure 6C). The results indicated that ECM modification (general ECM or Ca/Ic-induced ECM) promoted angiogenesis of the SIS scaffolds compared with that detected in the case of the raw SIS scaffolds. Moreover, the scaffolds implanted with BMSCs further promoted angiogenesis. The Ca/Ic-SIS scaffolds with BMSCs had the highest level of neovascularization among all groups.

## Discussion

In the past several decades, biomaterial-based bone grafts, including hydroxyapatite (HA) and collagens, have been used to mimic the microstructure of natural bone for tissue engineering (Gong and Niklason, 2008; Shakouri-Motlagh et al., 2017). However, the disadvantages of these grafts, such as the lack of osteoinduction and angiogenesis capacity, limit their clinical application (Shakouri-Motlagh et al., 2017). Compared with single or synthetic materials, ECM-based scaffolds with bone mimetic niches have attracted higher attention. Our previous study developed an excellent bone mimetic scaffold under the guidance of osteoblasts on SIS cotreated with icariin and calcium. Transplantation of the scaffolds in a mouse calvarial defect model induced approximately 75% new bone formation after 8 weeks via the BMP signaling pathway. The morphology, components, mechanical strength, and cellular activities *in vitro* were also characterized. Based on these findings, we applied the bone mimetic ECM scaffold with BMSCs to an ectopic bone formation model to further characterize the scaffold in the present study, discovering a better potential, including immune response, scaffold degradation, ectopic osteogenesis, and angiogenesis. All these capabilities can provide additional contributions to bone regeneration.

Immune cells, including neutrophils, mast cells, dendritic cells, T cells, monocytes, and macrophages, play a pivotal role in the regulation of anti-inflammatory effects of the immune system (Luster et al., 2005; Lee et al., 2019). Implantation of biomaterials into the defect area results in a complex inflammatory response and positively or negatively influences the bone healing process (Taraballi et al., 2018). At the initial stage, inflammation is desirable due to its key role in the recruitment of multiple cells and strong paracrine effects on skeletal-associated cells (i.e., osteoblasts, endothelial cells, and mesenchymal cells). However, over reactions of immune response labels implanted biomaterials as foreign to the body, leading to the blockade of therapeutic efficacy (Carnicer-Lombarte et al., 2021). For example, implantation of traditional metal screws (ZEK100) elicits a high inflammatory response and bone volume loss (Reifenrath et al., 2013). A number of studies attempted to modulate the immune response of the implants by employing biological molecules as stimulatory engineered microenvironments (Chen et al., 2018). Unlike synthetic scaffolds, natural ECM scaffolds present better potential to overcome the biological barriers posed by the immune system (Taraballi et al., 2018). Among these scaffolds, decellularized ECM from tissues or cells has been shown to positively influence the immune response and to promote bone regeneration (García-García and Martin, 2019; Rothrauff and Tuan, 2020).

SIS is a natural acellular matrix that has been extensively used for wound healing and tissue remodeling in the clinic with no immunogenicity. Ansaloni et al. investigated the immune response to a SIS implant in human patients (Ansaloni et al., 2007). The authors reported that SIS elicited an antibody response within a short time after the implantation, which was decreased later. No clinical rejection, wound infection, hernia recurrence, or other complications occurred during the 2-years follow-up. Kim et al. compared the innate immune response of the PLGA- and SIS-based scaffolds implanted in rats, and the results indicated considerably lower post-implantation effects in the case of the SIS-based scaffolds than that in the case of the PLGA-based scaffolds (Kim et al., 2007). In addition to immunogenicity, the results of the present study demonstrated that the SIS-based scaffolds (raw SIS and ECM-modified SIS) inhibited the immune response induced by ectopic implantation. The levels of inflammatory cells (monocytes and lymphocytes) were increased at the initial stage after the implantation and decreased earlier in the SIS-based groups compared with those in the sham group (Figure 1), which might be contributed by macrophage polarization. Several studies have reported that tissue regeneration induced by ECM scaffolds was associated with the promotion of a timely switch from pro-inflammatory (M1) to anti-inflammatory (M2) macrophages (Tidball and Villalta, 2010; Brown et al., 2012; LoPresti and Brown, 2018). In partial-thickness defect rats model, Brown et al. found that acellular ECM biomaterials promoted regeneration with a predominantly M2 type immune response, while biomaterials with cellular component resulted deposition of dense connective tissue and/or scarring with a predominantly M1 type response (Brown et al., 2009). Even though, the mechanism of SIS effects on immune response was rarely explored.

The new generation of biomaterials for tissue engineering involves the development of biodegradable components with excellent biocompatibility. ECM-based biomaterials, including SIS, and fulfill these requirements. Preclinical evaluation and clinical use suggested rapid degradation of SIS after the implantation, and this material can be replaced by the host tissue due to functional and histological similarities to the normal tissue (Gilbert et al., 2007). Our previous studies also demonstrated the degradation and reutilization of SIS in a mouse calvarial defect model (Li et al., 2017a; Li et al., 2018). The results of histological staining indicated the thinner layer of the SIS scaffolds, and the fibers of SIS were able to merge into the newly formed bone. However, for bone tissue engineering, artificial implants should provide sufficient mechanical strength until new bone is formed. On the one hand, SIS from the soft tissue lacks mechanical strength. On the other hand, rapid degradation *in vivo* leads to insufficient mechanical support and negatives effects on the patient’s activities, which limits clinical application. Thus, strategies to increase mechanical strength and postpone the degradation of SIS are critical for the application of SIS in bone regeneration. Our previous study generated an osteogenic and mineralized scaffold under the guidance of osteoblasts on SIS cotreated with icariin and calcium to improve its mechanical strength (Li et al., 2018). In the present study, the generated Ca/Ic-SIS scaffold was further proven to reduce the degradation speed of the SIS scaffold, which was able to provide longer mechanical support and facilitate bone regeneration (Figure 2).

Angiogenesis always accompanies osteoblast differentiation and bone formation and plays a critical role in bone formation (Deckers et al., 2000). Abundant studies have shown that BMSCs can promote angiogenesis, which makes them an ideal cell type for engineering of vascularized tissues (Ghajar et al., 2006; Ghajar et al., 2010). Zhuet al. investigated the effect of BMSCs on angiogenesis in a rat model of smoke inhalation injury, and the findings indicated that systemic transplantation of ^60^Co γ-ray-preconditioned BMSCs promotes angiogenesis through the Notch signaling pathway (Zhu et al., 2015). Then, several studies demonstrated specific matrix microenvironment can further promote the angiogenesis ability of BMSCs (Santhakumar et al., 2014; Zhang et al., 2014). Zhang et al. loaded VEGF and BMP-2 in silk scaffolds and implanted them subcutaneously in nude mice or in the rabbit skull defects (Zhang et al., 2014). They found that VEGF and BMP-2 induced the homing of tail vein injected BMSCs to the engineered scaffolds and promoted the differentiate of BMSCs into endothelial cells and osteogenic cells (Zhang et al., 2014). Santhakumar et al. developed a cardiac fibroblast-derived ECM, which was demonstrated to enhance angiogenesis better than BMSCs-derived ECM (Santhakumar et al., 2014). Protein composition of both ECMs were analyzed and angiogenesis associated proteins were mainly occurred in the cardiac fibroblast-derived ECM (Santhakumar et al., 2014). Our previous studies focused on cell derived ECMs (Li et al., 2018; Li et al., 2019; Li et al., 2020) and demonstrated optimized osteoblast-ECM promoted proliferation, osteogenesis, and stemness maintenance of BMSCs (Li et al., 2019). Proteomic analysis of osteoblast-ECM showed 24 ECM proteins took parts in angiogenesis, including CXCL12, THBS1, SPARC, ANGPTL4, and MMP14. Here, we further demonstrated SIS scaffolds ornamented by specific osteoblast-ECM (Ca/Ic-ECM) promoted angiogenesis *in vivo* after subcutaneous transplantation, compared with the effects of the raw SIS and general ECM-SIS scaffolds. Moreover, BMSCs seeded on the Ca/Ic-SIS scaffolds induced the highest levels of new vessel formation ratio (area %). These results indicated that interactions between BMSCs and Ca/Ic-ECM further promoted angiogenesis, which contributed to bone regeneration.

## Conclusion

In conclusion, the present study discovered multiple potential effects of generated bone mimetic scaffolds (Ca/Ic-SIS) in an ectopic osteogenesis model, including immune response, scaffold degradation, ectopic bone formation, and angiogenesis. As natural ECM biomaterials, the SIS scaffolds with or without ECM modification demonstrated low immunogenicity and reduced immune response caused by surgical operation at an early stage. ECM modification delayed the degradation of the SIS scaffolds, especially when the scaffolds were modified by Ca/Ic-ECM. Moreover, two kinds of ECM-modified SIS (ECM-SIS and Ca/Ic-SIS) were demonstrated to promote ectopic bone formation, and incorporation of BMSCs further promoted new bone formation in the Ca/Ic-SIS group. The Ca/Ic-SIS scaffolds demonstrated the greatest enhancement of angiogenesis, especially after the incorporation of BMSCs. These data and the results of our previous studies indicated that ECM modification under specific conditions is a potent strategy to improve the performance of multiple original materials in bone tissue engineering, which can be further promoted by incorporation of BMSCs.

## Data Availability

The original contributions presented in the study are included in the article/Supplementary Material, further inquiries can be directed to the corresponding authors.
